# The Autotransporter BpaB Contributes to the Virulence of *Burkholderia mallei* in an Aerosol Model of Infection

**DOI:** 10.1371/journal.pone.0126437

**Published:** 2015-05-20

**Authors:** Shawn M. Zimmerman, Frank Michel, Robert J. Hogan, Eric R. Lafontaine

**Affiliations:** 1 Department of Infectious Diseases, University of Georgia College of Veterinary Medicine, Athens, Georgia, United States of America; 2 Department of Veterinary Biosciences and Diagnostic Imaging, University of Georgia College of Veterinary Medicine, Athens, GA, United States of America

## Abstract

*Burkholderia mallei* is a highly pathogenic bacterium that causes the zoonosis glanders. Previous studies indicated that the genome of the organism contains eight genes specifying autotransporter proteins, which are important virulence factors of Gram-negative bacteria. In the present study, we report the characterization of one of these autotransporters, BpaB. Database searches identified the *bpaB* gene in ten *B*. *mallei* isolates and the predicted proteins were 99-100% identical. Comparative sequence analyses indicate that the gene product is a trimeric autotransporter of 1,090 amino acids with a predicted molecular weight of 105-kDa. Consistent with this finding, we discovered that recombinant bacteria expressing *bpaB* produce a protein of ≥300-kDa on their surface that is reactive with a BpaB-specific monoclonal antibody. Analysis of sera from mice infected with *B*. *mallei* indicated that animals produce antibodies against BpaB during the course of disease, thus establishing production of the autotransporter *in vivo*. To gain insight on its role in virulence, we inactivated the *bpaB* gene of *B*. *mallei* strain ATCC 23344 and determined the median lethal dose of the mutant in a mouse model of aerosol infection. These experiments revealed that the *bpaB* mutation attenuates virulence 8-14 fold. Using a crystal violet-based assay, we also discovered that constitutive production of BpaB on the surface of *B*. *mallei* promotes biofilm formation. To our knowledge, this is the first report of a biofilm factor for this organism.

## Introduction

Autotransporter proteins (AT) form one of the largest class of virulence factors in Gram-negative organisms and perform important functions in pathogenesis including flocculation [1, 2], formation of biofilms [3, 4], complement resistance [5, 6], host cell adhesion and entry [7–9], intracellular motility and replication [10, 11], cytotoxicity [12], and lipolytic activity [13–15]. These molecules share 4 common structural characteristics: a signal sequence (leader peptide), an N-terminal passenger domain, a C-terminal transporter domain, and a helical region of ~ 40 amino acids (aa) that links the passenger and transporter domains. The passenger domain is surface-exposed and specifies the biological function of the AT, while the transporter domain consists of hydrophobic β-strands and anchors the protein to the outer membrane (OM). Depending on the structure of the transporter domain, AT are classified as conventional or oligomeric [16–21]. Conventional AT have a large C-terminus of ~300 aa that form 10–12 antiparallel β-strands and are produced as monomers. In contrast, oligomeric AT have a short C-terminus (~70 aa) specifying only 4 β-strands and are typically produced as trimers.

After synthesis, AT are targeted to the inner membrane (IM) via their signal sequence and are secreted into the periplasm through the Sec translocase pathway [22]. Approximately 10% of AT possess a leader peptide with an Extended Signal Peptide Region (ESPR), which has been proposed to interact with the YidC IM protein integrase to slow translocation and prevent the accumulation of misfolded AT in the periplasm [22]. The molecular basis by which AT are secreted and displayed on the bacterial surface once they reach the periplasmic side of the OM is still under investigation. One proposed mechanism, the hairpin model, entails that AT self-secrete by inserting the C-terminal transporter domain into the OM to form a porin-like channel that is used to secrete the N-terminal passenger domain [16, 23]. Another model proposes that AT parasitize the β-barrel assembly machinery (Bam) complex, which assembles integral membrane proteins in the OM, thus transporting the AT to the cell surface [22].

Given their function in pathogenesis and overall structure, AT are excellent targets for developing medical countermeasures (MCM) against pathogenic organisms. A significant portion of AT (passenger domain) is readily accessible for recognition by the immune system as it is exposed on the bacterial surface. Additionally, AT perform key roles in virulence and targeting them may interfere with disease progression. Many reports have demonstrated the efficacy of AT-based countermeasures. For instance, a vaccine containing the AT cytotoxin Pta of *Proteus mirabilis* has been shown to elicit antibodies that neutralize the cytotoxicity of Pta for bladder cells *in vitro* and reduce bacterial loads as well as pathology in a mouse model of urinary tract infection [24]. Antibodies against *Haemophilus influenzae* Hap block adherence to epithelial cells *in vitro* and reduce nasopharyngeal colonization *in vivo* [25, 26]. Moreover, the AT adhesins *Bordetella pertussis* Pertactin and *Neisseria meningitidis* NadA are components of licensed vaccines against whooping cough (Daptacel, Infanrix, Boostrix, Adacel) and meningitis (Bexsero), respectively.

*Burkholderia mallei* is a Gram-negative coccobacillus for which developing MCM is desirable. The organism causes the zoonosis glanders, which predominantly affects equids, and is endemic to regions of Africa, South and Central America, the Middle East, and Asia [27–34]. Humans are generally infected with *B*. *mallei* through contact with tissues or body fluids of infected animals and the clinical manifestations include myalgia, fever, fatigue, lymphadenopathy, pneumonia, and dissemination of the agent to target tissues (spleen, liver, kidney) where it forms purulent lesions [33–35]. The recommended two-phase treatment process entails the use of the β-lactams ceftazidime and meropenem (intensive phase), followed by administration of Trimethoprim-Sulfamethoxazole (TMP-SMX) and the β-lactam Amoxicillin mixed with the β-lactamase inhibitor clavulanic acid (co-amoxiclav) for several weeks (eradication phase) [36].

The genome of *B*. *mallei* has been reported to encode 6 oligomeric AT gene products [37]. Of these, BoaA (adhesin, [38]), BimA (intracellular motility protein, [11]) and BpaC (adhesin, [39]) have been functionally characterized. The genome of the closely-related bacterium *Burkholderia pseudomallei* specifies these 6 AT gene products and all were recently studied to establish their biological roles as well as contribution to virulence [40]. One of these, named BpaB (locus tag number BP106B_I2046), was shown to be involved in adherence to human lung cells, which is a key step in pathogenesis of the agent because it leads to colonization [40]. In the present study, we characterized the *bpaB* ortholog of *B*. *mallei* and evaluated its contribution to virulence in a mouse model of aerosol infection.

## Materials and Methods

### Strains, plasmids, tissue culture cell lines, and growth conditions

Strains and plasmids are described in Table 1. *Burkholderia mallei* was cultured at 37°C using Brucella medium (BD) supplemented with 5% (vol/vol) glycerol. When indicated, antibiotics were added at the following concentrations: 7.5 μg/mL Polymyxin B (MP Biomedicals), 5 μg/mL kanamycin (MP Biomedicals), 7.5 μg/mL zeocin (Life Technologies). *Burkholderia mallei* bacteria used to inoculate mice were cultured on agar plates and suspended in PBS to the indicated concentration, as previously reported [41]. Aliquots of the bacterial suspension were immediately spread onto agar plates to determine the number of colony forming units (CFU) in the inoculum. *Escherichia coli* was cultured at 37°C using Low Salt Luria Bertani (LSLB) agar (Teknova) supplemented with 15 μg/mL chloramphenicol, 50 μg/mL kanamycin, 100 μg/mL ampicillin (Sigma-Aldrich), or 50 μg/mL zeocin, where applicable. The cell lines A549 (human type II alveolar epithelium; ATCC CCL85) and J774A.1 (murine macrophages; ATCC TIB-67) were cultured as described elsewhere [14, 38].

**Table 1:** Strains and plasmids.

Strain or plasmid	Description	Source or reference
**Strains**		
***B*. *mallei***		
**ATCC 23344**	Wild-type strain; Polymyxin B resistant, zeocin and kanamycin sensitive	[42]
***bpaB* KO**	Isogenic *bpaB* mutant strain of ATCC 23344; resistant to Polymyxin B and zeocin, kanamycin sensitive	This study
***E*. *coli***		
**EPI300**	Cloning strain	epicenter Illumina
**S17**	Strain used for conjugational transfer of plasmids pBHR1ΔDra, pBpaB and pKASbpaB.zeo to *B*. *mallei*; sensitive to Polymyxin B	[43]
**TUNER**	Production strain used for purification of His- and GST-tagged BpaB proteins	EMD Millipore
**Plasmids**		
**pBHR1**	Broad host range cloning vector; contains chloramphenicol and kanamycin resistance markers	MoBiTec Molecular Biotechnology
**pBHR1ΔDra**	pBHR1 containing a 339-nt deletion in the chloramphenicol resistance marker; confers resistance only to kanamycin	This study
**pBpaB**	pBHR1ΔDra containing the *B*. *mallei* ATCC 23344 *bpaB* gene; kanamycin resistant	This study
**pCC1**	Cloning vector; contains chloramphenicol resistance marker	epicenter Illumina
**pCCbpaB**	pCC1 containing the *B*. *mallei* ATCC 23344 *bpaB* gene; chloramphenicol resistant	This study
**pCCbpaB.zeo**	pCCbpaB in which a 1.4-kb fragment internal to the *bpaB* ORF is replaced with a 0.4-kb zeocin resistance cassette; confers resistance to chloramphenicol and zeocin	This study
**pKAS46**	Mobilizable suicide plasmid; contains kanamycin resistance marker	[44]
**pKASbpaB.zeo**	pKAS46 containing the insert from pCCbpaB.zeo; confers resistance to kanamycin and zeocin	This study
**pEM7/ZEO**	Source of the zeocin resistance marker	Life Technologies
**pETcoco-1**	Protein production vector; chloramphenicol resistant	EMD Millipore
**pHisBpaB**	pETcoco-1 producing BpaB residues 57–984 joined to an N-terminal His-tag; chloramphenicol resistant	This study
**pGEX4T-2**	Protein production vector; ampicillin resistant	GE Healthcare Life Sciences
**pGSTBpaB**	pGEX4T-2 producing BpaB residues 57–984 joined to an N-terminal GST-tag; ampicillin resistant	This study

### Recombinant DNA methodology

Standard molecular biology methods were performed as outlined by others [45]. Genomic DNA was purified using the Easy-DNA Kit (Life Technologies). Plasmid DNA was purified using the QIAprep Spin Miniprep kit (Qiagen).

The broad host range plasmid pBHR1 was digested with *Dra*I, excised from an agarose gel, purified with the High Pure PCR Product Purification kit (Roche Applied Science), and ligated in order to delete a 339-nucleotide (nt) fragment internal to the chloramphenicol resistance marker. The resulting plasmid, designated pBHR1ΔDra, was sequenced to verify that the deletion was successfully engineered. The plasmid was also tested to confirm that it no longer confers resistance to chloramphenicol.

Platinum *Pfx* DNA Polymerase (Life Technologies) was used in all PCR experiments as per the manufacturer’s recommended conditions. A DNA fragment of 3,516-nt containing the *bpaB* gene was amplified from the genome of *B*. *mallei* ATCC 23344 with primers P1 (5’-GCC TCG GCA AAT AAA TTT CAA TTG-3’) and P2 (5’-GTC GGA GAG CAC GTA TGC ATT GAA-3’). The amplicon was cloned in the vector pCC1 using the CopyControl PCR cloning kit (epicenter Illumina), producing plasmid pCCbpaB. The latter was digested with *BamH*I, and a 3,529-nt fragment containing the *bpaB* gene was purified from agarose gel slices, treated with the End-It DNA End Repair Kit (epicenter Illumina), and subcloned into the *Dra*I site of pBHR1ΔDra to yield plasmid pBpaB.

The plasmid pCCbpaB was digested with the enzymes *Rsr*II and *Asc*I to remove a 1,454-nt fragment internal to the *bpaB* ORF, treated with the End-It DNA End Repair Kit, and ligated with a blunt 0.4-kilobases (kb) zeocin resistance cassette to yield plasmid pCCbpaB.zeo. This plasmid was restricted with *BamH*I, and a 2.5-kb fragment corresponding to the *bpaB* ORF disrupted by the insertion of the zeocin resistance cassette was gel-purified, treated with the End-It DNA End Repair Kit, and subcloned into the *EcoR*V site of the suicide vector pKAS46, producing plasmid pKASbpaB.zeo.

A PCR product specifying aa 57 to 984 of the *bpaB* ORF was generated from *B*. *mallei* ATCC 23344 genomic DNA with oligonucleotides P3 (5’-CCC AAG CTT GGC ACG GAT AAC GTC TA-3’; *Hind*III site underlined) and P4 (5’-GGT TAA TTA AAG GAC CGC ATC GGT CG-3’; *Pac*I site underlined). This amplicon was purified, digested with *Hind*III and *Pac*I, and ligated in-frame with the N-terminal His-tag of vector pETcoco-1. The resulting plasmid was designated pHisBpaB. A similar approach was used to engineer plasmid pGSTBpaB, which encodes the same portion of the *bpaB* gene product joined to an N-terminal Glutathione-*S*-Transferase (GST) tag. The DNA fragment was amplified with primers P5 (5’-CGG GAT CCG GCA CGG ATA ACG TCT A-3’; *Bam*HI site underlined) and P6 (5’-CCG CTC GAG TGA CCG CAT CGG TCG C-3’; *Xho*I site underlined) and cloned in the vector pGEX4T-2.

Plasmids were sequenced to verify that PCR did not introduce mutations resulting in aa substitutions in the *bpaB* gene product. Restriction endonucleases and T4 DNA ligase were purchased from New England BioLabs, Inc.

### Construction of a *B*. *mallei* ATCC 23344 *bpaB* isogenic mutant strain

The plasmid pKASbpaB.zeo was introduced in the *E*. *coli* strain S17 by electroporation and subsequently transferred into *B*. *mallei* ATCC 23344 by conjugation, as previously reported [38, 46]. Upon conjugation, *B*. *mallei* colonies were first selected for resistance to Polymyxin B (to prevent growth of *E*. *coli* S17) and zeocin (to select strains containing the disrupted copy of *bpaB* in their genome). These potential mutants were then tested for sensitivity to kanamycin to identify strains that do not contain the suicide vector pKAS46 integrated in their genome. Lastly, colonies were screened by PCR with primers P7 (5’-CGC TCG ATA CCC ACG TTT AT-3’) and P8 (5’-GCG ATG CAG ATG CGT ATA GA-3’). The primers yielded a PCR product of 7.6-kb in the parent strain and a smaller amplicon of 6.6-kb in the *bpaB* KO mutant (data not shown). This 1-kb difference in size is consistent with deletion of the aforementioned 1.4-kb *Rsr*II-*Dra*I fragment internal to the *bpaB* ORF and insertion of the 0.4-kb zeocin resistance marker in its place. The PCR product from the mutant strain was sequenced to verify proper allelic exchange and successful disruption of *bpaB*.

In order to perform complementation experiments, the plasmids pBHR1ΔDra and pBpaB were electroporated in *E*. *coli* S17 and subsequently transferred into the *B*. *mallei bpaB* KO mutant by conjugation. Conjugants were selected for resistance to zeocin (specified by the isogenic mutation in the genome of strain *bpaB* KO), kanamycin (specified by pBHR1ΔDra and pBpaB), and Polymyxin B (to prevent growth of *E*. *coli* S17).

### Nucleotide sequence and bioinformatics analyses

Plasmids and purified PCR fragments were sequenced at the University of Michigan Sequencing Core (http://seqcore.brcf.med.umich.edu). Sequencher 5 (Gene Codes Corporation) was used to analyze chromatograms. Assembled contigs were analyzed with Vector NTI (Life Technologies) and online tools available at the ExPASy Bioinformatics Resource Portal (http://www.expasy.org).

### Experiments with human lung epithelial cells and murine macrophages

Plate-grown bacteria (40-hr for *B*. *mallei*, 20-hr for *E*. *coli*) were used for all experiments. Adherence and intracellular survival assays were performed as previously reported [38, 39, 47, 48]. Cells were infected with bacteria at a multiplicity of infection of 100 (adherence) and 10 (intracellular survival). Duplicate assays were performed on at least 3 occasions. Statistical analyses were performed using the Mann-Whitney test (GraphPad Prism 6 software), and *P* values < 0.05 are reported as statistically significant.

### Crystal violet-based biofilm assay

This procedure was modified from a protocol published by Pearson *et al* [49]. Plate-grown bacteria (20-hr for both *B*. *mallei* and *E*. *coli*) were suspended in broth to a density of 10^7^ CFU/mL and 100 μL of these suspensions were seeded into quadruplate wells of polyvinyl chloride (PVC) round bottom microplates (Corning). These plates were then incubated statically at 37°C (24-hr for *E*. *coli*, 48-hr for *B*. *mallei*). Following this, broth was removed from the wells and replaced with 200 μL of Ham’s F12 medium (cellgro) supplemented with 0.035% (wt/vol) crystal violet (Sigma-Aldrich). After 30-min at room temperature, the crystal violet staining mixture was removed and the wells were washed with deionized water. A 200 μL volume of methanol was added to each well and the plate was incubated for 30 min. Well contents were transferred to a new microplate and the absorbance at a wavelength of 570 nm, which is indicative of bacteria forming biofilms on PVC and stained with crystal violet, was measured spectrophotometrically. Statistical analyses were performed using the Mann-Whitney test (GraphPad Prism 6 software), and *P* values < 0.05 are reported as statistically significant.

### Antigen preparations and analysis

Total membrane proteins and sarkosyl-insoluble fractions containing OM proteins were obtained as described by Carlone *et al* [50]. The method used to prepare whole cell lysates and perform western blot experiments are described elsewhere [47]. The plasmids pHisBpaB and pGSTBpaB were electroporated in the *E*. *coli* strain TUNER for the purpose of producing and purifying His- and GST-tagged BpaB proteins, respectively. Both proteins were extracted from inclusion bodies and purified under denaturing conditions as previously outlined by our laboratory [14, 51]. Purified capsular polysaccharides (CPS) from the *Burkholderia pseudomallei* LPS^-^ mutant strain MB100 [52] were kindly provided by Donald E. Woods at the University of Calgary.

To obtain polyclonal antibodies directed against BpaB, purified His-tagged BpaB was emulsified in Freund’s adjuvants (Sigma-Aldrich) and administered to female BALB/c mice (Frederick National Laboratory for Cancer Research) as reported by Lafontaine and colleagues [53]. Serum antibodies were demonstrated to recognize BpaB by western blot using purified GST-tagged BpaB (data not shown). The BpaB-specific monoclonal antibody #4 (BpaB-MAb#4) was generated by fusing splenocytes from a vaccinated mouse with Sp2/mIL6 cells (ATCC CRL 2016). The fused cells were plated in methylcellulose medium containing hypoxanthine, aminopterin, and thymidine using a ClonaCell HY kit per the manufacturer’s specifications (Stemcell Technologies). Hybridomas secreting antibodies specific for BpaB were identified by ELISA.

For ELISA, duplicate wells of Immulon 2HB plates (Thermo Scientific Nunc) were coated with purified antigens (CPS, GST-tagged BpaB) overnight at 4°C. Unbound antigens were removed by washing wells with 1X wash solution (KPL) and the wells were then filled with 10% BSA Diluent/Blocking solution (KPL) diluted to 0.5% with PBS and supplemented with 3% (wt/vol) dry milk (blocking buffer). After incubation at room temperature for 1-hr, the wells were washed with 1X wash solution and probed for 1-hr at room temperature with primary antibodies (serum from mice that survived aerosol infection with *B*. *mallei* ATCC 23344, serum from mice immunized with His-tagged BpaB, culture supernatants from hybridomas producing monoclonal antibodies) diluted in blocking buffer. After this incubation, the wells were washed with 1X wash solution and incubated at room temperature with goat anti-mouse secondary antibodies conjugated to Alkaline Phosphatase diluted in blocking buffer. After washing off the excess secondary antibodies with 1X wash solution, 100 μL of *p*NPP solution (KPL) was added to wells. Color development, indicative of antibody binding to antigen, was measured by determining the absorbance of well contents at a wavelength of 405 nm.

### Immunofluorescence labeling

Production of BpaB on the bacterial surface was visualized by immunofluorescence microscopy as outlined by Balder *et al* [38]. Briefly, plate-grown bacteria (40-hr for *B*. *mallei*, 20-hr for *E*. *coli*) were fixed with 4% (wt/vol) paraformaldehyde, spotted onto glass slides, probed with α-BpaB polyclonal antibodies, and incubated with a goat α-mouse antibody labeled with Alexa Fluor 488 (Life Technologies) and the nucleic acid dye DAPI (Life Technologies). Slides were examined by microscopy using a Nikon Eclipse Ti confocal system. A minimum of 5 random fields was assessed on at least 2 separate occasions for each strain.

Production of BpaB on the bacterial surface was measured by flow cytometry as described by Lipski and colleagues [14]. Plate-grown bacteria (40-hr for *B*. *mallei*, 20-hr for *E*. *coli*) were fixed with 4% (wt/vol) paraformaldehyde, probed with α-BpaB polyclonal antibodies, and incubated with a goat α-mouse antibody labeled with Alexa Fluor 488. Labeled bacteria were analyzed using a BD LSR II flow cytometer.

### Aerosol infection experiments

Female BALB/c mice (14–28 weeks of age at the time of infection) were purchased from Frederick National Laboratory for Cancer Research. The animals were anesthetized by injecting a dose of 250 mg/kg of 2, 2, 2 tribromoethanol (TBE, Sigma-Aldrich) intraperitoneally. Once anesthetized, mice were inoculated intratracheally with 50 μL of bacterial suspensions using a Microsprayer model I-1C (PennCentury) as previously reported by our laboratory [39, 41]. Infected animals were monitored daily. Food and water were provided *ad libitum*. Humane end-points were strictly observed. Mice exhibiting signs of moderate to severe discomfort were euthanized. This was accomplished by anesthetizing the animals with TBE followed by cervical dislocation, in accordance with the AVMA Guidelines on euthanasia. Tissues (lungs and spleen) were aseptically collected, homogenized, serially diluted, and plated on agar medium to calculate bacterial loads. Survival data were analyzed using the Kaplan-Meier method, and the LD_50_ values were calculated according to Reed and Muench [54].

### Compliance and animal research ethic statements

All experiments with live *B*. *mallei* were performed inside a Class II Biosafety Cabinet in a BSL3 laboratory and in compliance with the rules and regulations of the U. S. Federal Select Agent Program. The University of Georgia’s Institutional Biosafety Committee (IBC) approved the experiments.

Animal experiments were carried out in strict accordance with the recommendations in the Guide for the Care and Use of Laboratory Animals of the National Institutes of Health. The University of Georgia’s Institutional Animal Care and Use Committee (IACUC) approved the experiments. All efforts were made to minimize animal suffering.

## Results

### Selected features of the *bpaB* genomic locus and gene product

**Fig 1:**
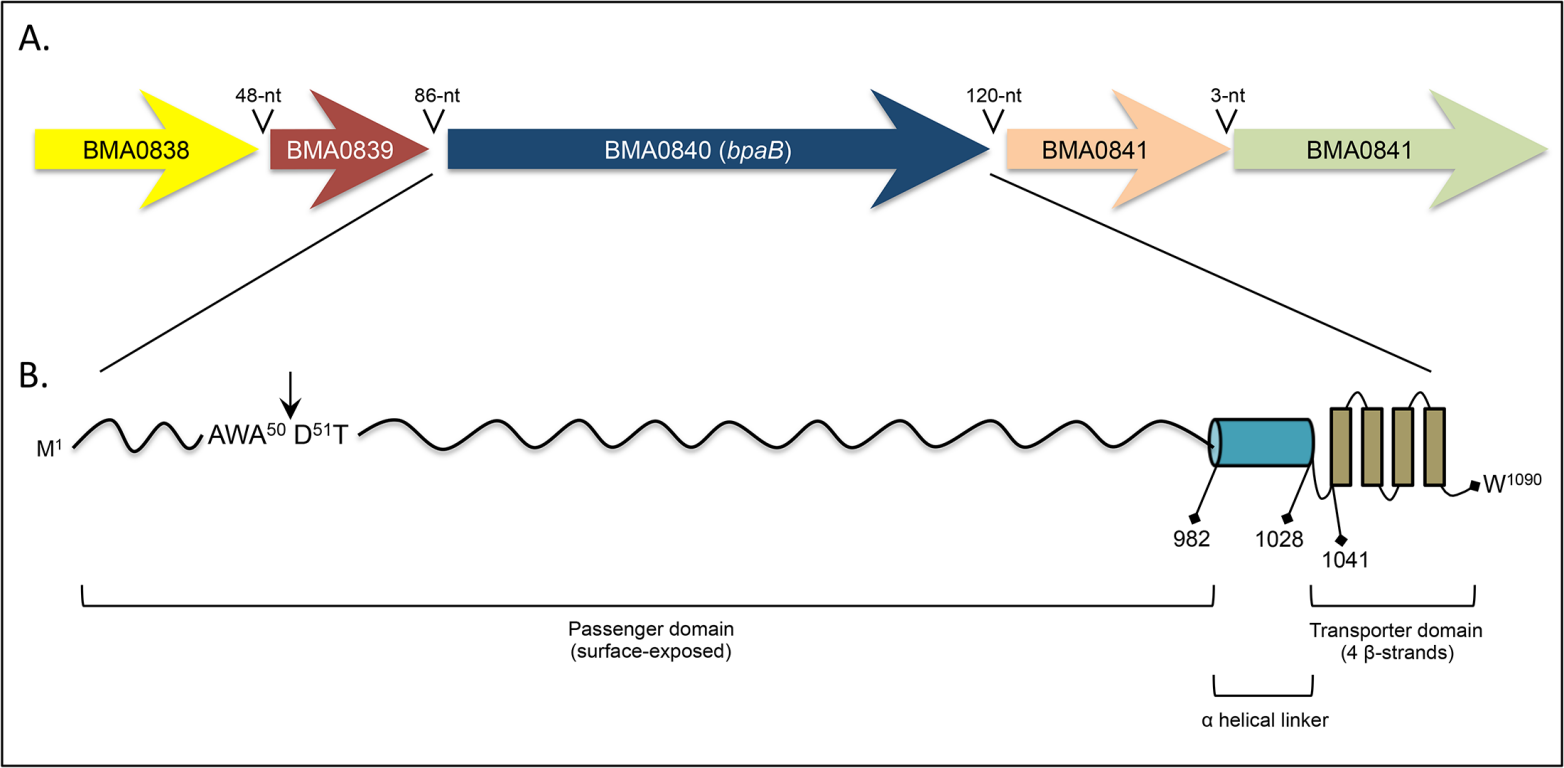
Schematic representation of the *B*. *mallei* ATCC 23344 *bpaB* genomic locus and gene product. Panel A: Colored block arrows represent the *bpaB* ORF and surrounding genes in chromosome I of the organism. The length of intergenic regions is shown above block arrows. Panel B: Different regions of the predicted BpaB protein are depicted with positions of residues defining selected domains. The C-terminal transporter domain and helical linker were identified using the PSIPRED server. The arrow represents the predicted signal sequence cleavage site, which was identified using the SignalP 4.1 server.

Comparative sequence analyses identified a *B*. *pseudomallei bpaB* ortholog on chromosome I of the *B*. *mallei* ATCC 23344 genome (locus tag number BMA0840). The ORF is 3,270-nt in length and predicted to specify a protein of 1,090 aa with a molecular mass of 105,486 (Fig 1, Table 2). Database searches with the NCBI Microbial Protein BLAST service also identified the *bpaB* gene in another 10 *B*. *mallei* (including ATCC 23344) and 29 *B*. *pseudomallei* strains. The predicted proteins were found to be highly-conserved among isolates of both species (99–100% identity). Interestingly, the genome of the closely-related bacterium *Burkholderia thailandensis*, which is a non-pathogenic environmental saprophyte [55–58], does not contain the *bpaB* gene. Potential ORFs were located upstream and downstream of *bpaB*, and these ORFs show similarities to a transcriptional regulator and the well-characterized OM protein OmpA, respectively (Fig 1A, Table 2).

**Table 2:** Selected features of the *bpaB* genomic locus of *B*. *mallei* ATCC 23344.

Locus tag number	Length of gene product and predicted MW	Description
**BMA0838**	231 aa, 26-kDa	Putative DNA-binding response regulator of the OmpR family (CDD number 223816, E-value of 2.31e-57).
**BMA0839**	80 aa, 9-kDa	Hypothetical protein, no conserved domain found or similarity to other proteins.
**BMA0840 (*bpaB*)**	1090 aa, 105-kDa	AT, 99% identical to *B*. *pseudomallei* 1026b BpaB [40].
**BMA0841**	223 aa, 23-kDa	Putative OmpA_C-like OM protein based on the high level of sequence similarity of residues aa 110–204 to CDD domain number 143586 (E-value of 4.22e-22).
**BMA0842**	509 aa, 56-kDa	Hypothetical protein. Residues 81–175 show sequence similarity with TPR domain (Tetratricopeptide repeat domain, CDD number 238112, E-value of 1.94e-10). Proteins containing this domain are often involved in protein-protein interactions.

As depicted in Fig 1B, the *B*. *mallei* ATCC 23344 *bpaB* gene product possesses conserved structural features of oligomeric AT. Analysis with the PSIPRED secondary structure prediction method revealed that the last 50 aa form 4 antiparallel β-strands, each connected by short loops of 4 residues. This potential membrane-anchoring transporter domain is preceded by a helical region of 46 residues (aa 982–1,028), and a putative signal sequence cleavage site was detected at the N-terminus between aa 50 and 51 using the SignalP 4.1 server. Sequence analysis of BpaB using the Entrez Conserved Domains Database (CDD) also identified several domains commonly associated with oligomeric AT (Table 3).

**Table 3:** Regions of BpaB with high level of sequence similarity to conserved domains in the NCBI Entrez Conserved Domain Database (CDD).

BpaB residues	E-value	CDD domain	CDD number	Description
**1–24**	8.25e-08	ESPR	257462	Extended Signal Peptide of Type V secretion system. Present at the N-terminus of the signal peptides of proteins belonging to the Type V secretion systems, including AT.
**108–251**	2.16e-3	LbR-like Superfamily	248061	Left-handed β-roll. This family contains a variety of domains with a left-handed β-roll structure including virulence factors and various other proteins such as the oligomeric AT UspA1 of *Moraxella catarrhalis* and YadA of *Yersinia enterocolitica*.
**346–439**	1.93e-12	LbR_YadA-like	240612	YadA-like, left-handed β-roll. This group contains the collagen-binding domain of the virulence factor and oligomeric AT YadA of *Y*. *enterocolitica*.
**330–1090**	1.9e-16	HIA	227614	Autotransporter adhesin [Intracellular trafficking and secretion/ Extracellular structures] first identified in the oligomeric AT Hia of *Haemophilus influenzae*.
**1014–1090**	9.64e-14	YadA_anchor	252233	YadA-like C-terminal region. Represents the C-terminal 120 aa of several oligomeric AT including *Y*. *enterocolitica* YadA, *M*. *catarrhalis* UspA2, *Haemophilus ducreyi* DsrA and *E*. *coli* Eib. The C-terminal 9 aa, consisting of alternating hydrophobic aa ending in F or W, comprise a targeting motif for the OM of the Gram-negative cell envelope. This region is also important for oligomerization.

### Production and functional analysis of the BpaB protein in *E*. *coli*

**Fig 2:**
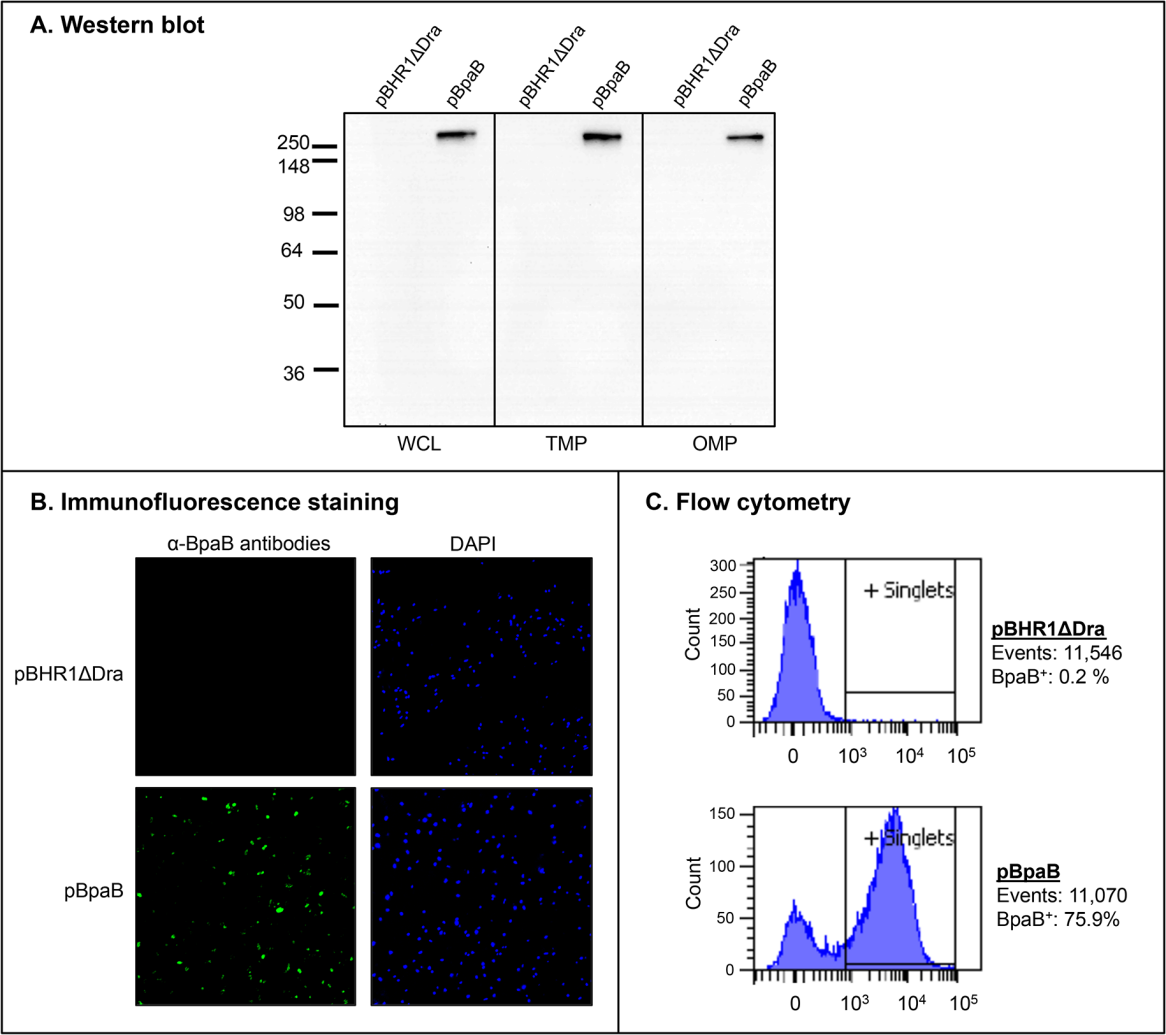
BpaB production by *E*. *coli* recombinant strains. Panel A: Equivalent amounts of whole cell lysates (WCL), total membrane proteins (TMP) and sarkosyl-insoluble fractions containing OM proteins (OMP) were resolved by SDS-PAGE, transferred to PVDF membranes and analyzed by western blot with the monoclonal antibody BpaB-MAb#4. Molecular weight markers are shown to the left in kilodaltons. Panel B: Non-permeabilized *E*. *coli* strains were fixed onto glass slides and fluorescently-labeled with DAPI (blue) and with α-BpaB polyclonal antibodies (green) as described in Materials and Methods. Bacteria were visualized by microscopy using a Nikon Eclipse Ti confocal system. Representative microscopic fields are shown. Panel C: Non-permeabilized *E*. *coli* strains were incubated with polyclonal antibodies against BpaB and fluorescently-labeled with a goat α-mouse antibody conjugated with the fluorochrome Alexa Fluor 488. Labeled bacteria were analyzed using a BD LSR II flow cytometer. The *x*-axis represents the level of fluorescence, and the *y*-axis corresponds to the particles counted in arbitrary units. The number of cells analyzed, and the percentage of those producing BpaB on their surface, is shown.

The data presented in Table 3 indicate that BpaB exhibits sequence and structural similarities to the adhesins *M*. *catarrhalis* UspA1 [59], *Y*. *enterocolitica* YadA [5], and *H*. *influenzae* Hia [60]. Hence, we hypothesized that BpaB mediates adherence to epithelial cells. In addition, previous work demonstrated that mutating the *bpaB* gene in *B*. *pseudomallei* reduces adherence to A549 human type II pneumocytes [40]. To determine whether BpaB mediates adherence to epithelial cells, the *bpaB* gene of *B*. *mallei* ATCC 23344 was cloned and expressed in the recombinant background of the nonadherent *E*. *coli* cloning strain EPI300. To verify protein production, whole cell lysates (WCL), total membrane proteins (TMP), and sarkosyl-insoluble fractions containing OM proteins (OMP) were prepared from *E*. *coli* EPI300 harboring the plasmid pBHR1ΔDra (control) or pBpaB (specifies *B*. *mallei* ATCC 23344 *bpaB*) and analyzed by western blot. Fig 2A shows that the BpaB-specific monoclonal antibody BpaB-MAb#4 reacts with an OMP of ~300-kDa in bacteria expressing the *bpaB* gene, which closely matches the predicted trimeric molecular weight of the gene product (315-kDa).

Immunofluorescence-labeling and microscopy examination of bacteria was used to establish that BpaB is displayed on the surface of *E*. *coli*. As shown in Fig 2B, bacteria carrying pBpaB are stained by α-BpaB polyclonal antibodies, whereas *E*. *coli* carrying the control plasmid pBHR1ΔDra is not. Labeling of nucleic acids with DAPI verified that comparable numbers of bacteria were examined. Surface-display of BpaB was confirmed by flow cytometry analysis of immunofluorescently-labeled cells. Fig 2C demonstrates that α-BpaB polyclonal antibodies caused a shift in fluorescence of *E*. *coli* carrying pBpaB, indicating that they bind to the surface of intact recombinant bacteria producing BpaB. As expected, α-BpaB antibodies did not bind to the surface of *E*. *coli* carrying the control plasmid. Of note, the BpaB-specific antibodies used in western blot, immunofluorescence microscropy, and flow cytometry experiments were raised against a purified recombinant His-tagged protein encompassing aa 57–984 of BpaB, which corresponds to the predicted surface-exposed passenger domain (Fig 1B).

**Fig 3:**
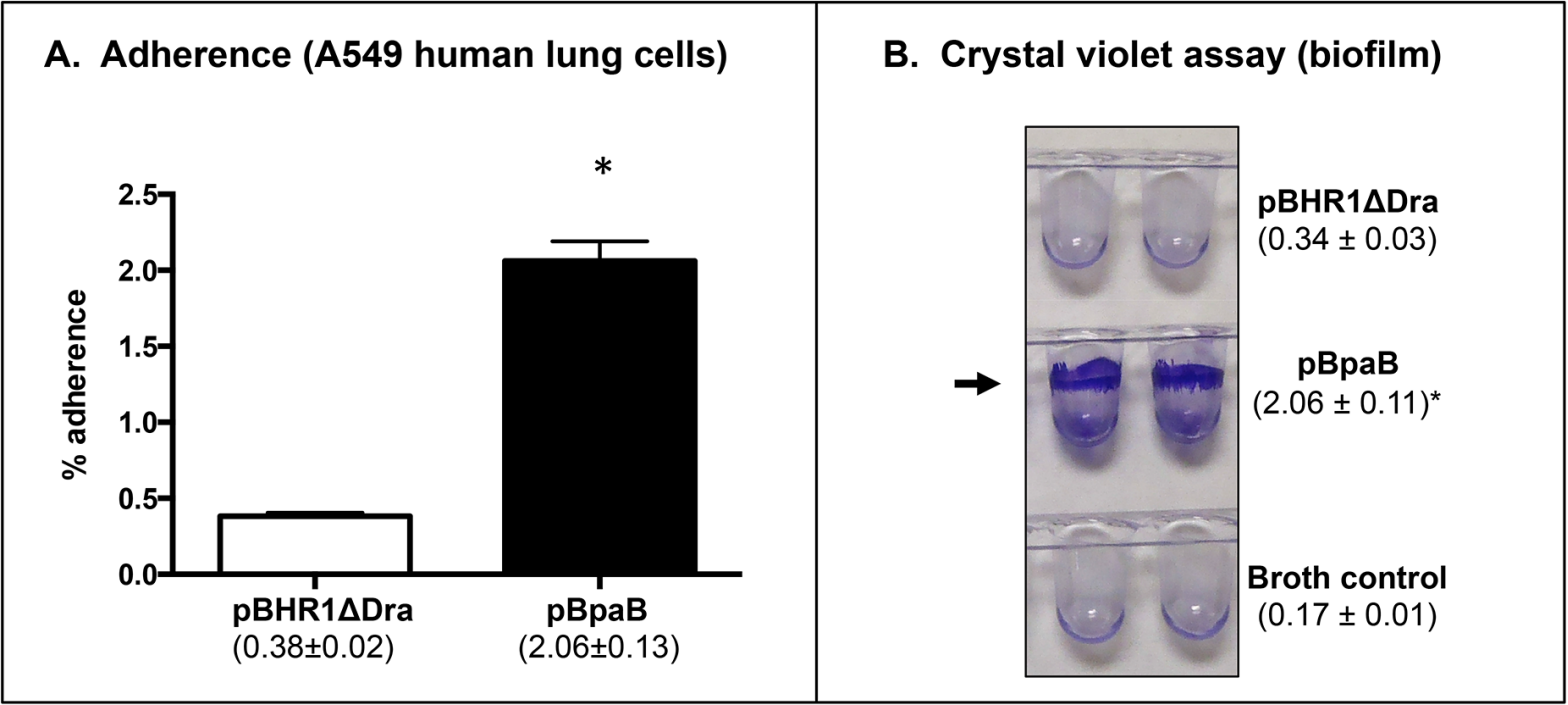
Adherence and biofilm assays with *E*. *coli* recombinant strains. Panel A: *E*. *coli* strains were incubated with epithelial cells for 30 min at 37°C. Following this, cells were washed to remove unbound bacteria, lysed, diluted, and spread onto agar plates to calculate the number of bound bacteria. The results are expressed as the mean percentage (± standard error) of inoculated bacteria attached to A549 cells. The values in parentheses show the actual percentage. Panel B: *E*. *coli* strains were cultured in the wells of PVC microplates, stained with crystal violet, washed with deionized water, and the wells were photographed. The arrow shows biofilm formation, which was quantitated by extracting crystal violet with methanol and measuring absorbance at 570 nm. The results are shown in parentheses and are expressed as the mean (± standard error) absorbance. Both panels: The asterisks indicate that the increase in adherence and biofilm formation of *E*. *coli* carrying pBpaB, compared to *E*. *coli* harboring pBHR1ΔDra, is statistically significant (*P* values < 0.05, Mann-Whitney test).

Quantitative adherence assays revealed that bacteria producing BpaB bind to A549 cells at levels 5-fold greater than bacteria carrying pBHR1ΔDra (Fig 3A). These data show that BpaB mediates adherence to airway cells. *Burkholderia mallei* is a serum-resistant facultative intracellular bacterium that replicates inside different types of eukaryotic cells. Furthermore, AT adhesins often perform additional functions including biofilm formation [49, 61], serum resistance [5, 59], invasion [7], and intracellular survival/replication [10]. Therefore, we tested whether *E*. *coli* producing BpaB form biofilms using a crystal violet staining assay. We discovered that recombinant bacteria carrying plasmid pBpaB form a ring-like pellicle located at the liquid-air interface, indicative of biofilm formation, whereas *E*. *coli* harboring the control plasmid pBHR1ΔDra did not (Fig 3B). In additional experiments, we also discovered that BpaB production by *E*. *coli* does not increase serum resistance, epithelial cell entry, or phagocytosis by/survival inside J774 murine macrophages (data not shown). Taken together, our data indicate that BpaB is a multifunctional oligomeric AT that mediates adherence to human lung cells and contributes to biofilm formation.

### Production and functional analysis of the BpaB protein in *B*. *mallei*

**Fig 4:**
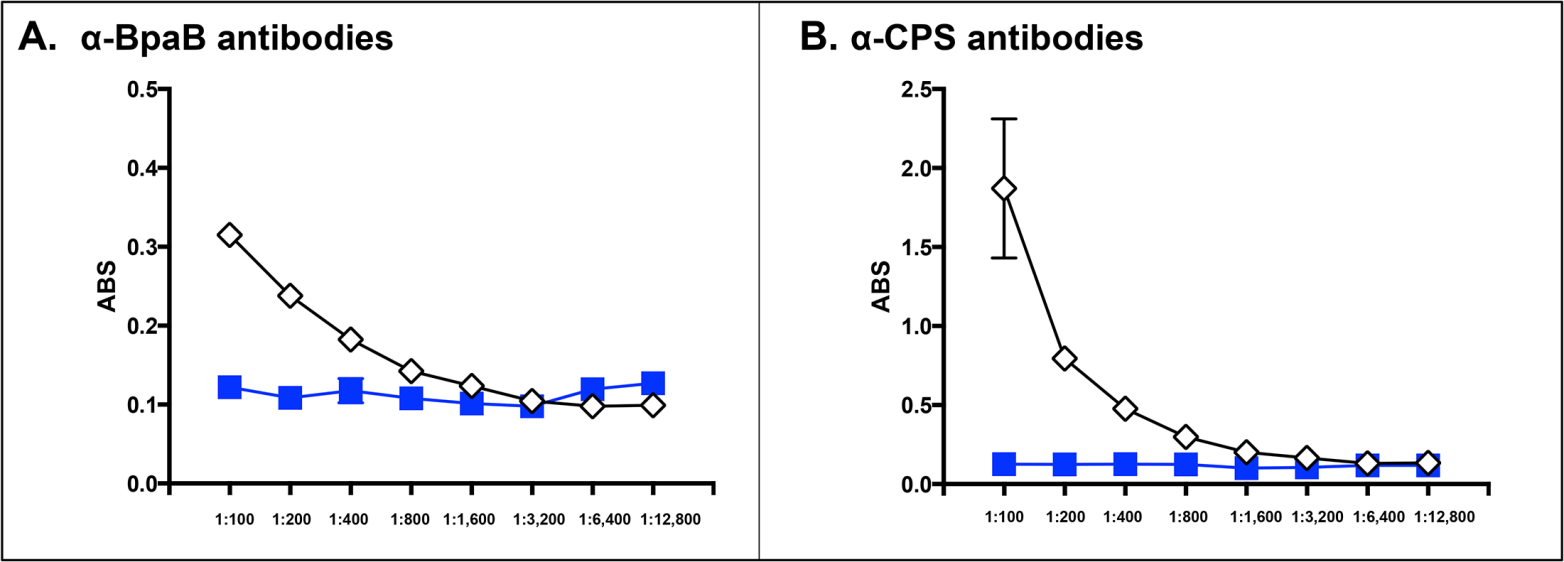
ELISA with sera from mice that survived aerosol challenge with *B*. *mallei*. Serum samples were serially diluted and placed in duplicate wells of plates coated with GST-tagged BpaB (panel A) and CPS (panel B). Goat α-mouse antibodies (light and heavy chains) conjugated to Alkaline Phosphatase were used as secondary antibodies. The y-axis shows absorbance at a wavelength of 405 nm, which is indicative of antibodies binding to antigens coating the plates. The x-axis represents serial two-fold dilutions of sera. The results are expressed the mean absorbance (± standard error). Open diamonds show sera from mice that survived challenge with *B*. *mallei* ATCC 23344. Blue squares represent sera from control mice that were inoculated with 50 μL of PBS using the MicroSprayer. These serum samples were generated in the context of a previously published study [41].

To investigate the function of the *bpaB* gene product in the native background of *B*. *mallei*, we constructed an isogenic mutant of strain ATCC 23344. Whole cell lysates were prepared from wild-type (WT) and mutant strains, and analyzed by western blot to verify lack of BpaB production in the mutant. However, α-BpaB antibodies did not react with protein preparations of the parent strain (or mutant, as expected). Other detection methods such as immunofluorescence-labeling and immunoprecipitation also failed to demonstrate BpaB production. These findings indicate that the AT is not produced at detectable levels under routine *in vitro* growth conditions. To determine whether BpaB is produced *in vivo*, we used ELISA to test serum samples from mice infected via the aerosol route with *B*. *mallei* ATCC 23344. Panel A in Fig 4 shows that mice produced antibodies against BpaB, with a reciprocal end-point titer of 400; serum from control mock-infected mice did not contain α-BpaB antibodies. As positive control, we tested serum samples for the presence of antibodies against capsular polysaccharides (CPS), which are known immunogenic molecules on the surface of *B*. *mallei* during infection [62]. As shown in Fig 4B, the reciprocal end-point titer of α-CPS antibodies in the serum of infected mice is 3,200.

**Fig 5:**
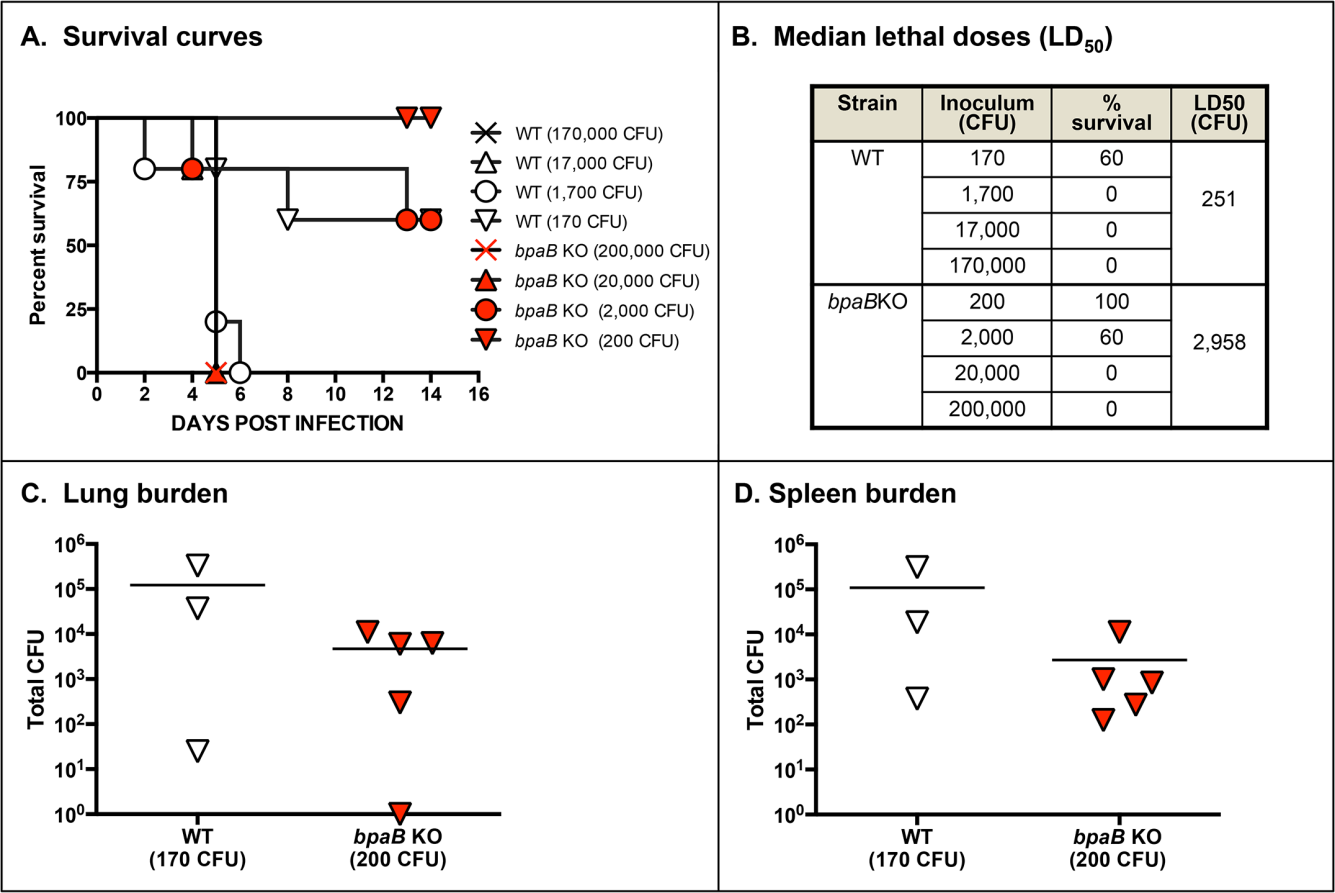
Median lethal dose determination of WT *B*. *mallei* and *bpaB* KO mutant strains. Mice were inoculated intratracheally using a Microsprayer device to aerosolize the indicated number of bacterial CFU directly into the lungs (n = 5 mice/dose). Animals were then monitored daily for clinical signs of illness and morbidity. Panel A: Survival curves. Panel B: Calculated LD_50_ values. Panels C and D: Tissues were collected from mice that survived challenge with 10^2^ CFU, homogenized, diluted, and spread on agar plates to determine bacterial loads. Symbols show data for individual animals.

To gain insight on the role of BpaB in virulence, we determined the median lethal dose of the mutant using a mouse model of aerosol infection. In these experiments, we also collected tissues from survivors and determined bacterial burden, which is indicative of *in vivo* fitness. We discovered that the *bpaB* mutation attenuates *B*. *mallei* virulence 12-fold (Fig 5A and 5B). We also found that at equivalent inoculating doses of 10^2^ CFU, bacterial loads in the lungs (Fig 5C) and spleen (Fig 5D) of survivors were comparatively reduced in mice infected with the mutant. This attenuation in virulence does not appear to be related to *in vitro* defects of the mutant in growth rate, serum resistance, epithelial cells adherence/invasion, phagocytosis by murine macrophages, or survival inside these immune cells (data not shown).

**Fig 6:**
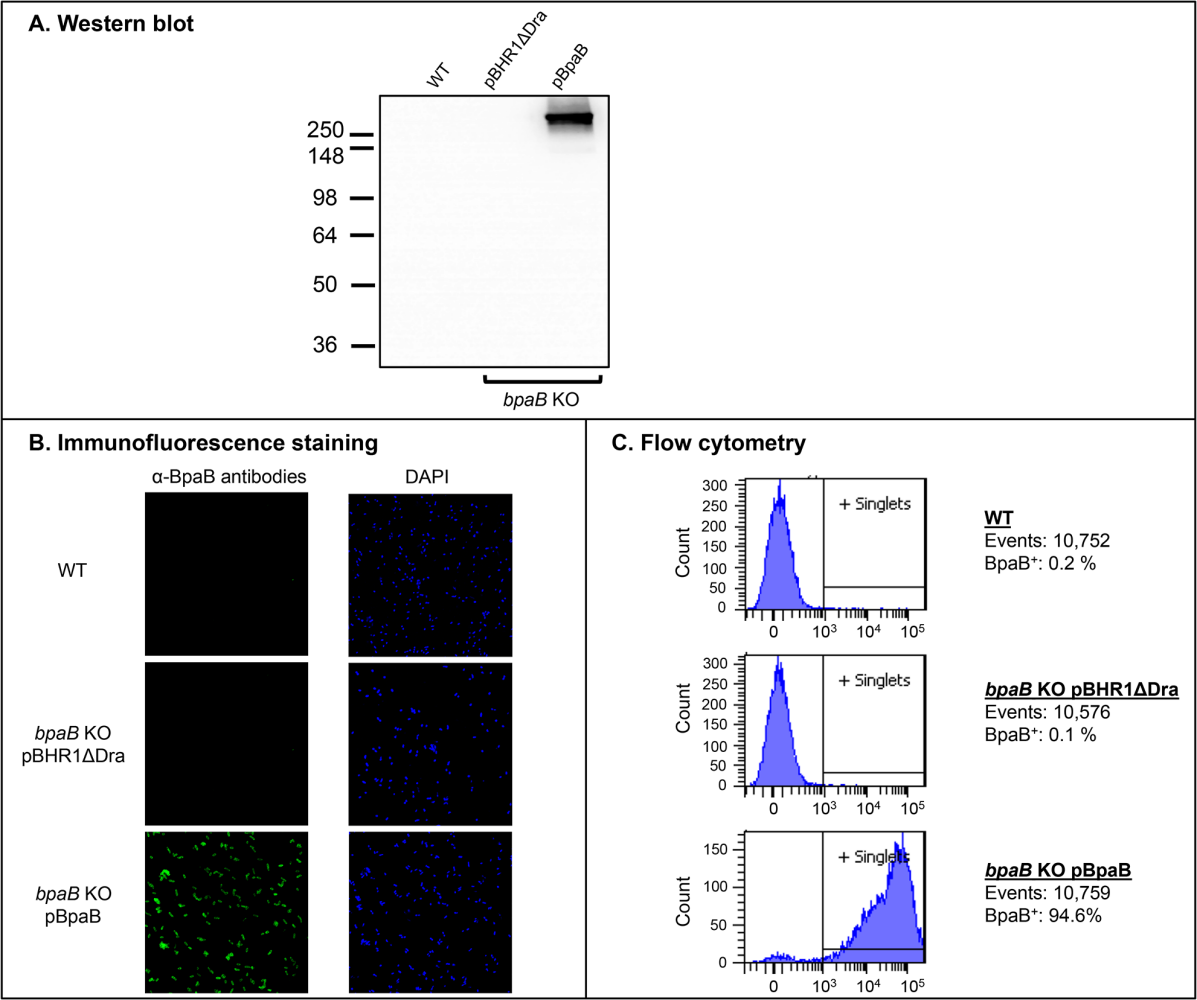
BpaB production by *B*. *mallei* recombinant and WT strains. Panel A: Equivalent amounts of whole cell lysate preparations were resolved by SDS-PAGE, transferred to PVDF membranes and analyzed by western blot with BpaB-MAb#4. Molecular weight markers are shown to the left in kilodaltons. Panel B: Non-permeabilized *B*. *mallei* strains were fixed onto glass slides and fluorescently-labeled with DAPI (blue) and with α-BpaB polyclonal antibodies (green) as described in Materials and Methods. Bacteria were visualized by microscopy using a Nikon Eclipse Ti confocal system. Representative microscopic fields are shown. Panel C: Paraformaldehyde-fixed *B*. *mallei* strains were incubated with polyclonal antibodies against BpaB and fluorescently-labeled with a goat α-mouse antibody conjugated with the fluorochrome Alexa Fluor 488. Labeled bacteria were analyzed using a BD LSR II flow cytometer. The *x*-axis represents the level of fluorescence, and the *y*-axis corresponds to the particles counted in arbitrary units. The number of cells analyzed, and the percentage of those producing BpaB on their surface, is shown.

**Fig 7:**
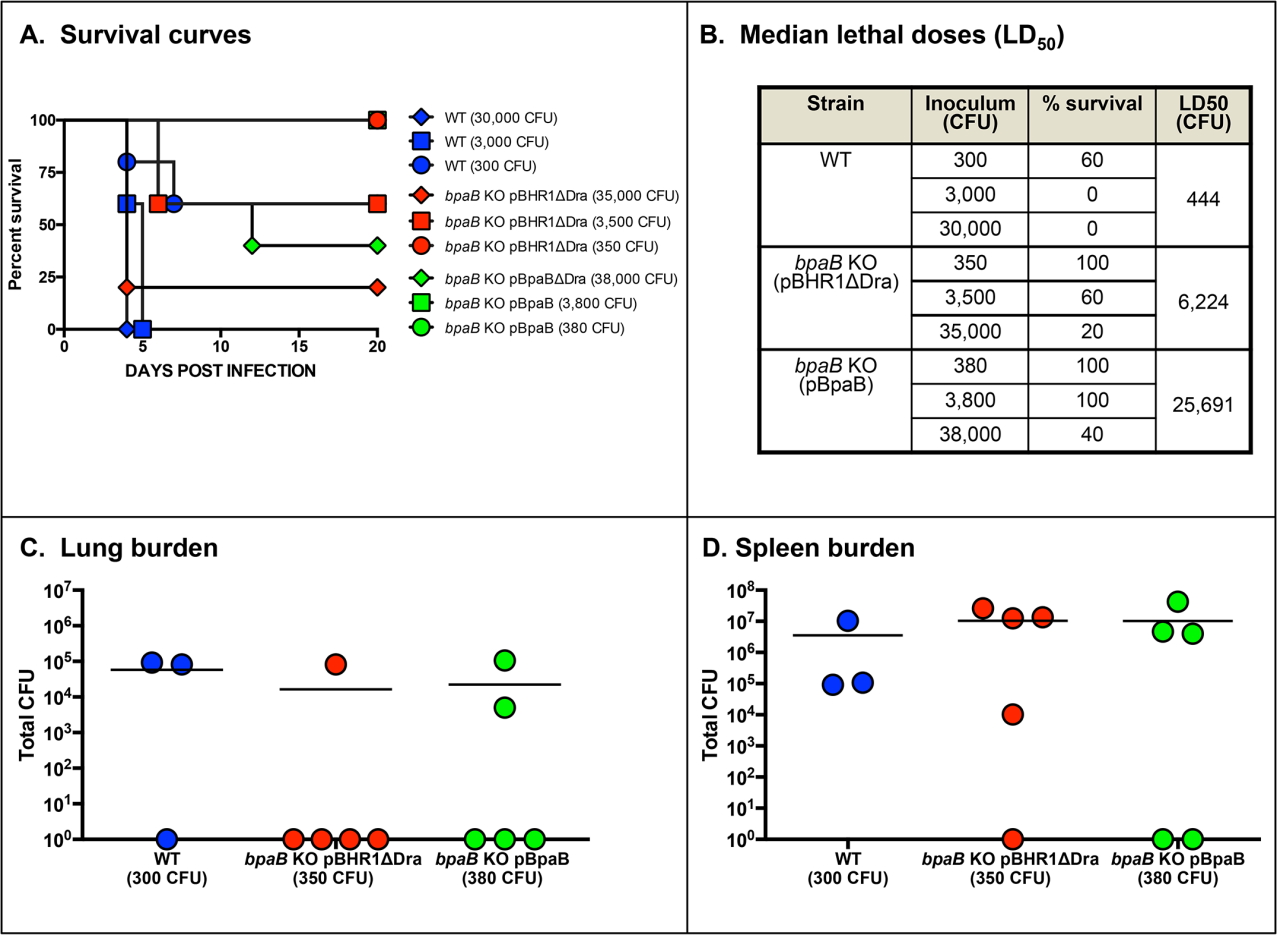
Median lethal dose determination of WT *B*. *mallei* and recombinant *bpaB* KO strains. Mice were inoculated intratracheally using a Microsprayer device to aerosolize the indicated number of bacterial CFU directly into the lungs (n = 5 mice/dose). Animals were then monitored daily for clinical signs of illness and morbidity. Panel A: Survival curves. Panel B: Calculated LD_50_ values. Panels C and D: Tissues were collected from mice that survived challenge with 10^2^ CFU, homogenized, diluted, and spread on agar plates to determine bacterial loads. Symbols show data for individual animals.

To further investigate the functional properties of BpaB and its contribution to virulence, we introduced plasmid pBpaB in the *bpaB* KO mutant. As shown in Fig 6, recombinant bacteria produced an antigen of the expected size (Panel A) on their surface (Panels B and C). Surprisingly, 2 independent experiments to determine the median lethal dose of the complemented mutant revealed that constitutive production of BpaB further attenuates virulence. The *bpaB* KO mutant carrying the control plasmid pBHR1ΔDra shows 8- and 14-fold reductions in LD_50_ values (Fig 7B and S1B Fig, respectively) when compared to WT *B*. *mallei*, which is consistent with the 12-fold attenuation in virulence measured for the mutant without the plasmid (Fig 5B). In contrast, the LD_50_ values of the mutant harboring plasmid pBpaB were 55- and 58-fold lower than those of the parent strain (Fig 7B and S1B Fig, respectively). At similar inoculating doses (~10^2^ CFU), bacterial loads in the lungs and spleen of survivors were slightly reduced in mice infected with the mutant compared to animals inoculated with WT *B*. *mallei* (Panels C and D in Fig 7 and S1 Fig). To verify that the reduced virulence phenotype of the *bpaB* KO strain producing BpaB is not due to an overall growth defect, we measured the replication of WT *B*. *mallei* alongside the *bpaB* KO mutant carrying plasmids pBHR1ΔDra and pBpaB in broth. We found that all strains grew at comparable rates (S2 Fig). These experiments also established that in the absence of antibiotic selection, both plasmids are stably maintained (data not shown).

**Fig 8:**
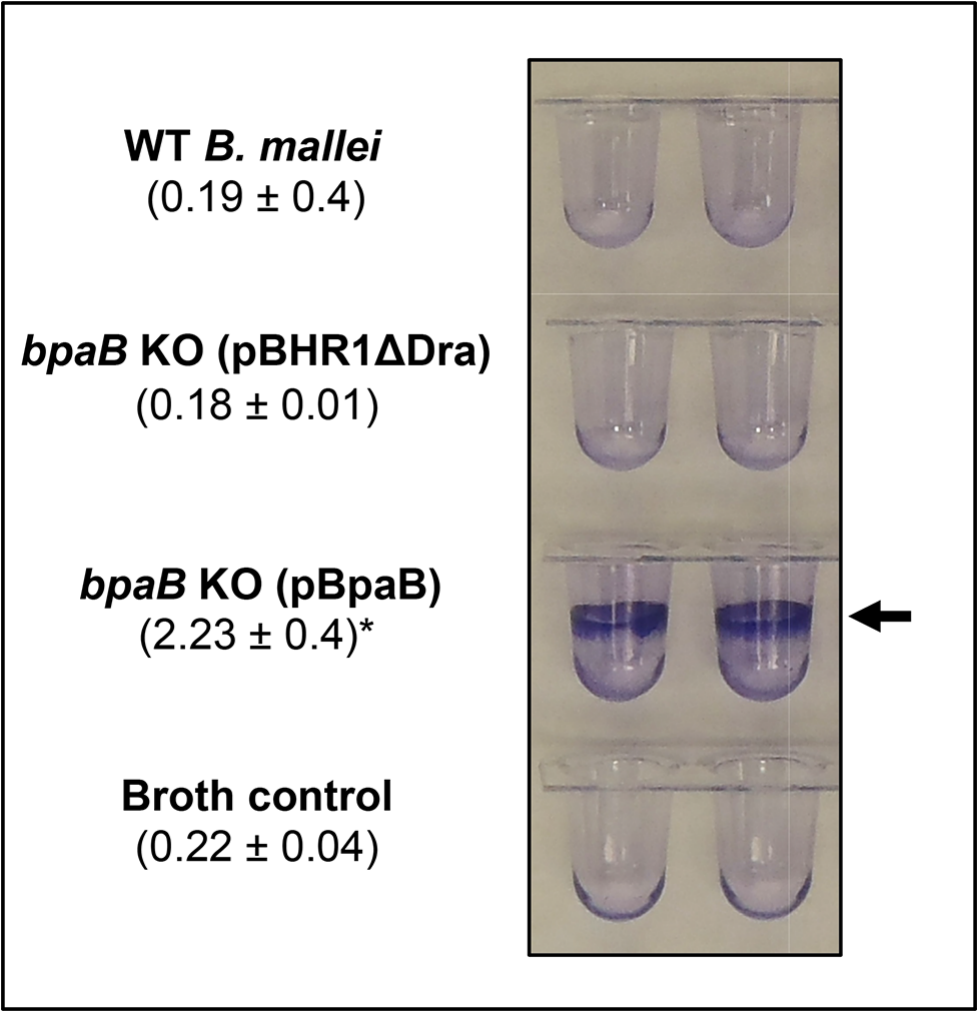
Biofilm assay with *B*. *mallei* recombinant and WT strains. *B*. *mallei* strains were cultured in the wells of PVC microplates, stained with crystal violet, washed with deionized water, and the wells were photographed. The arrow shows biofilm formation, which was quantitated by extracting crystal violet with methanol and measuring absorbance at 570 nm. The results are shown in parentheses and are expressed as the mean (± standard error) absorbance. The asterisk indicates that the increase in biofilm formation by bacteria carrying pBpaB is statistically significant (*P* values < 0.05, Mann-Whitney test).

Because biofilm and adherence assays with recombinant *E*. *coli* bacteria indicate that BpaB production increases biofilm formation and adherence to A549 cells (Fig 3), we compared the ability of WT *B*. *mallei* and the *bpaB* KO strain carrying plasmids pBHR1ΔDra and pBpaB to form biofilm and attach to epithelial cells. Consistent with the *E*. *coli* data, we found that BpaB production confers the mutant with the ability to form biofilms (Fig 8). The mutant carrying the control plasmid pBHR1ΔDra and WT *B*. *mallei*, which does not express BpaB under the growth conditions used to perform experiments, did not form ring-like pellicles at the liquid-air interface. Quantitative adherence assays indicated that all 3 strains adhered to A549 cells at comparable levels (data not shown).

## Discussion

This study demonstrates that BpaB is a surface-located oligomeric AT that promotes the formation of biofilms and contributes to the virulence of *B*. *mallei* in a model of aerosol infection. We show that BpaB is conserved among sequenced *B*. *mallei* isolates, is produced *in vivo*, and elicits the production of antibodies during infection. Hence, BpaB displays many properties of an excellent target for developing countermeasures.

We discovered that *B*. *mallei* ATCC 23344 does not produce detectable amounts of BpaB when cultured under routine laboratory conditions. This does not appear to be a strain- or species-specific phenomenon. Western blot analysis of lysates from *B*. *mallei* ATCC 10399 (China 5), *B*. *pseudomallei* 1026b, and *B*. *pseudomallei* K96243 also show lack of reactivity with α-BpaB polyclonal and monoclonal antibodies (data not shown), even though the genome of these strains specify a WT *bpaB* gene (locus tag numbers BMA10399_E0436, BP106B_I2046, BPSL2063, respectively). Published microarray data for the organisms are consistent with our findings and suggest precise regulation of *bpaB*. The gene is not appreciably expressed *in vitro* by *B*. *mallei* isolates [63] and is not upregulated by restricting iron concentrations in the culture medium [64]. In addition, *bpaB* expression is not detected in the liver of Syrian golden hamsters two days after infection with *B*. *mallei* ATCC 23344 [42]. Ooi and colleagues recently measured expression of *B*. *pseudomallei* K96243 genes under 82 conditions, many of which mimicking exposures the organism encounters in the environment or in an infected host [65]; *bpaB* is 1 of 468 genes that does not exhibit detectable expression under any of the conditions tested.

BpaB is the fourth *B*. *mallei* oligomeric AT reported as not being produced under routine laboratory growth conditions. BoaA [38] and BpaC [39], both AT adhesins for airway cells, and the intracellular motility protein BimA [66, 67] are the others. The *bimA* gene is under the control of a two-component regulatory system consisting of the sensor histidine-kinase protein VirA and the DNA response regulator VirG. The *virAG* genes are contiguous to *bimA* in the Cluster 1 Type VI Secretion System T6SS-1 on chromosome II of *B*. *mallei* ATCC 23344, and are up-regulated following internalization of the agent into phagocytic cells [67, 68]. It has also been shown that constitutive overproduction of VirAG activates *bimA* transcription under routine *in vitro* culture conditions [67]. Interestingly, a putative DNA binding response regulator of the OmpR family is located upstream of *bpaB* (see BMA0838 in Fig 1 and Table 2). Open reading frames specifying DNA response regulators were also identified adjacent to the *boaA* and *bpaC* genes in the *B*. *mallei* ATCC 23344 genome (data not shown). Based on these observations, it is tempting to speculate that *bpaB* expression is under the control of the BMA0838 gene product. The BpaB-specific antibody reagents as well as recombinant strains developed in this study will facilitate testing this hypothesis.

While not produced under routine laboratory growth conditions, BpaB is produced during the course of aerosol infection of mice as indicated by the presence of BpaB-specific antibodies in serum samples after resolution of acute infection (Fig 4). Sera from horses with experimental glanders have also been shown to contain high antibody titers against BpaB [69]. These results are especially compelling because horses are the natural host and reservoir for *B*. *mallei* and are arguably the most relevant surrogate to study glanders. Using protein microarray technology, Varga *et al* demonstrated that serum from a human patient with glanders also contain increased levels of α-BpaB antibodies [70]. Our animal experiments showing that a mutation in *bpaB* attenuates virulence (Figs 5 and 7, S1 Fig) substantiate these observations and underscore the potential usefulness of BpaB as a target for developing countermeasures. The AT is produced during infection and contributes to pathogenesis by *B*. *mallei*. Hence, targeting BpaB may interfere with disease progression.

The analysis of recombinant bacteria constitutively producing BpaB suggests that the AT contributes to pathogenesis by promoting biofilms. Using a crystal violet staining assay, we discovered that *E*. *coli* and *B*. *mallei* producing BpaB both form ring-like biofilm structures on polyvinyl at the air-liquid interface of static broth cultures (Figs 3B and 8). To our knowledge, this is the first report of a *B*. *mallei* gene product directly participating in biofilm formation. Quantitative attachment assays revealed that BpaB production significantly increases adherence of *E*. *coli* to A549 human lung cells (Fig 3A). Conversely, we found that constitutive production of the AT does not have a measurable effect on *B*. *mallei* adherence (data not shown). Likewise, the mutation in the *bpaB* gene does not impair binding of strain ATCC 23344 to A549 cells. One possible explanation for this lack of effect is that other adhesins produced by the *bpaB* KO mutant provide a high background of adherence. For instance, BoaA [38] and BpaC [39] have been shown to mediate binding of *B*. *mallei* ATCC 23344 to A549 cells. The organism also produces a Type IV pilus [71], which is a common adherence factor of Gram-negative bacteria [72]. It is also possible that the increased adherence measured when BpaB is produced in the heterologous genetic background of *E*. *coli* is a result of biofilm formation. Continued investigation of the molecular mechanism by which *B*. *mallei* regulates BpaB production and a detailed structure-function analysis of the AT will clarify its role in adherence and biofilm formation. Of note, Lazar Adler *et al* have reported that *B*. *pseudomallei* also produces an AT directly involved in biofilms, BbfA (locus tag number BPSS1439) [73]. The *bbfA* ortholog of *B*. *mallei* is BMAA0810, not BpaB, and has yet to be functionally characterized.

The most surprising result of our study is the discovery that constitutive production of BpaB by the *bpaB* KO mutant strain does not restore virulence, but instead further attenuates the organism (Fig 7 and S1 Fig). While this finding seems counter-intuitive, there are precedents in the literature for such a phenomenon. For example, constitutive production of the *Cryptococcus neoformans* adhesin Cfl1 [74], the *Salmonella enterica* flagellum [75], and the *Yersinia pestis* F1 capsule [76] have been reported to substantially attenuate the virulence of these organisms. In fact, overproduction of bacterial surface-associated molecules is an emerging technique for developing novel live attenuated vaccines dubbed Attenuating Gene Expression (AGE) [77]. The molecular basis for attenuation is not fully understood and appears to vary between model systems. In the case of the *Salmonella* flagellum, overproduction of the flagellar apparatus has been shown to disrupt the structural integrity of the bacterial membranes, which in turn facilitates clearance of the agent by innate immune mechanisms. On the other hand, constitutive production of Cfl1 by *C*. *neoformans* appears to impact pathogenicity through increased and untimely cell adhesion. The mechanism by which constitutive production of BpaB attenuates virulence is currently being investigated.

Taken together, our data demonstrate that BpaB is a virulence factor and that precise regulation of production is central to its role in the pathogenesis of *B*. *mallei*. Identification of the specific signals that modulate BpaB production as well as the host-related cues influencing these pathways will provide novel insights in the complex biology and virulence of the organism, and will yield useful information for the development of MCM and diagnostics for glanders.

## Supporting Information

S1 Fig Median lethal dose determination of WT *B*. *mallei* and recombinant *bpaB* KO strains. Mice were inoculated intratracheally using a Microsprayer device to aerosolize the indicated number of bacterial CFU directly into the lungs (n = 5 mice/dose). Animals were then monitored daily for clinical signs of illness and morbidity. Panel A: Survival curves. Panel B: Calculated LD_50_ values. Panels C and D: Tissues were collected from mice that survived challenge with 10^2^ CFU, homogenized, diluted, and spread on agar plates to determine bacterial loads. Symbols show data for individual animals.
(TIF)

S2 Fig Growth rates of WT *B*. *mallei* and recombinant *bpaB* KO strains in liquid cultures. Plate-grown bacteria (40-hr) were suspended in broth to an optical density at wavelength 600 nm (ABS_600nm_) of ~ 0.1. Following this, suspended bacteria were incubated at 37°C and the optical density of cultures was measured at the indicated time intervals. Strains were tested on at least 3 separate occasions. Representative experiments are shown.
(TIF)
