# Supplementary figures and images for: The Autotransporter BpaB Contributes to the Virulence of Burkholderia mallei in an Aerosol Model of Infection
Source: PLoS One. 2015 May 20;10(5):e0126437. doi: 10.1371/journal.pone.0126437 (PMC4438868; doi:10.1371/journal.pone.0126437)

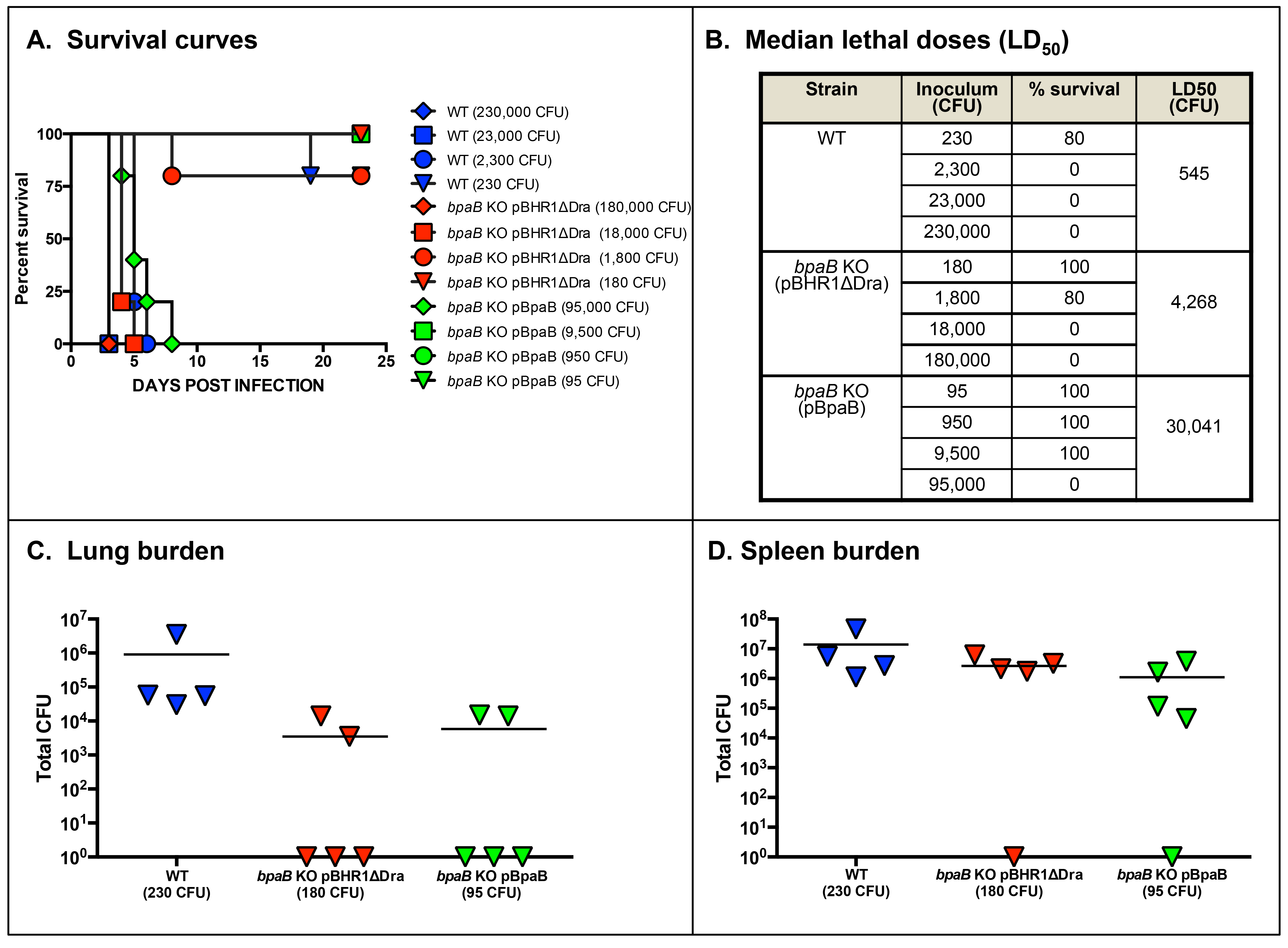

Supplement: S1 Fig — Mice were inoculated intratracheally using a Microsprayer device to aerosolize the indicated number of bacterial CFU directly into the lungs (n = 5 mice/dose). Animals were then monitored daily for clinical signs of illness and morbidity. Panel A: Survival curves. Panel B: Calculated LD50 values. Panels C and D: Tissues were collected from mice that survived challenge with 102 CFU, homogenized, diluted, and spread on agar plates to determine bacterial loads. Symbols show data for individual animals. (TIF) [file pone.0126437.s001.tif]

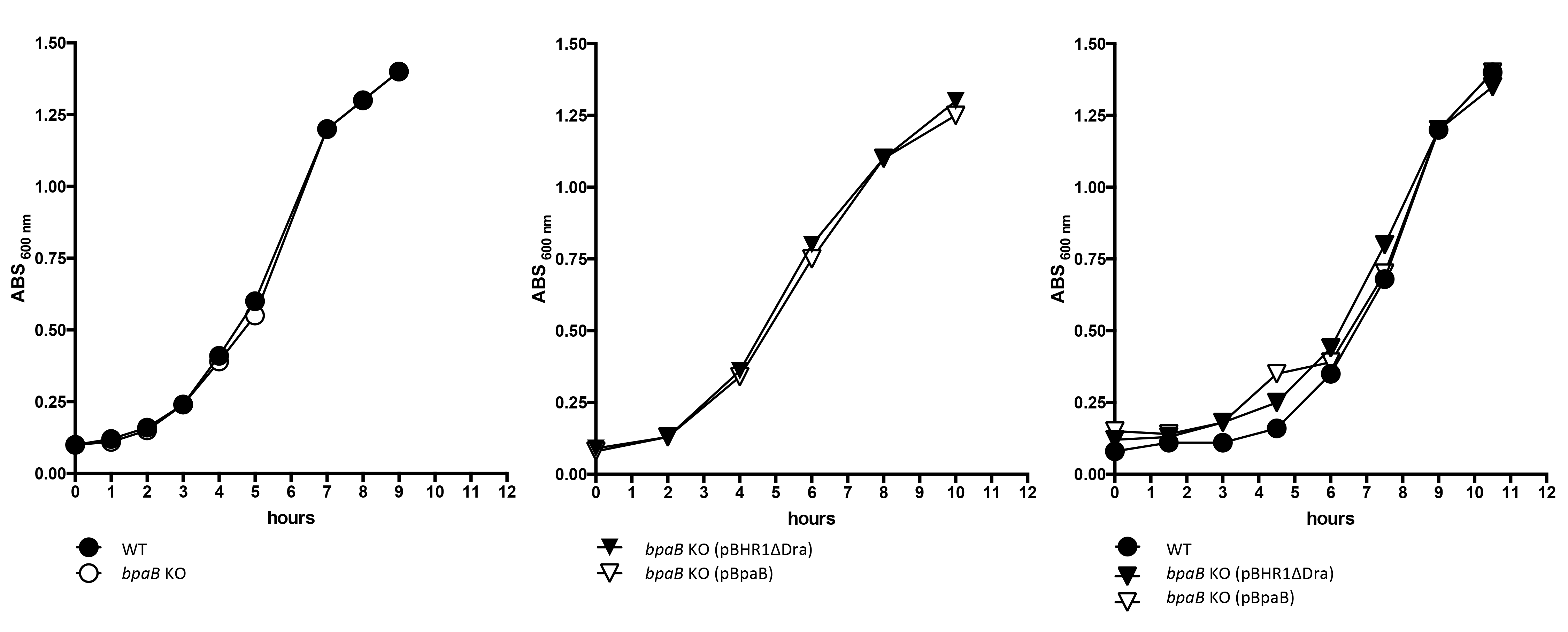

Supplement: S2 Fig — Plate-grown bacteria (40-hr) were suspended in broth to an optical density at wavelength 600 nm (ABS600nm) of ~ 0.1. Following this, suspended bacteria were incubated at 37°C and the optical density of cultures was measured at the indicated time intervals. Strains were tested on at least 3 separate occasions. Representative experiments are shown. (TIF) [file pone.0126437.s002.tif]
